# Incidence and risk factors for intussusception among children in northern Israel from 1992 to 2009: a retrospective study

**DOI:** 10.1186/1471-2431-14-218

**Published:** 2014-08-31

**Authors:** Khitam Muhsen, Eias Kassem, Sigalit Efraim, Sophy Goren, Dani Cohen, Moshe Ephros

**Affiliations:** 1 Department of Epidemiology and Preventive Medicine, School of Public Health, Sackler Faculty of Medicine, Tel Aviv University, Ramat Aviv, Tel Aviv 69978, Israel; 2 Department of Pediatrics, Hillel Yaffe Medical Center, Hadera, Israel; 3 Department of Pediatrics, Carmel Medical Center and the Faculty of Medicine, Technion-Israel Institute of Technology, Haifa, Israel

**Keywords:** Intussusception, Risk factors, Surgery, Ethnicity, Pediatrics

## Abstract

**Background:**

Determining the background incidence of intussusception is important in countries implementing rotavirus immunization. Rotavirus immunization was introduced into the routine infant immunization program in Israel during late 2010. Incidence and risk factors for intussusception were examined in children aged less than five years between 1992 and 2009.

**Methods:**

Data were collected from medical records of children hospitalized due to intussusception (N = 190), and from control children (N = 295), at Carmel and Hillel Yaffe hospitals in northern Israel.

**Results:**

The average annual incidence of intussusception in Jewish and Arab children aged less than five years was estimated at 36.1 (95% CI 17.0-76.5) vs. 23.2 per 100,000 (95% CI 9.3-57.9); for infants less than 12 months of age- 128.1 (95% CI 53.0-309.6) vs. 80.1 (95% CI 29.1-242.6) per 100,000. The risk of intussusception was higher in infants aged 3–5 months: OR 5.30 (95% CI 2.11-13.31) and 6–11 months: OR 2.53 (95% CI 1.13-5.62) when compared to infants aged less than 3 months; in those living in low vs high socioeconomic communities: OR 2.81 (95% CI 1.45-5.43), and in children with recent gastroenteritis: OR 19.90 (95% CI 2.35-168.32) vs children without recent gastroenteritis. Surgical reduction was required in 23.2%. The likelihood of surgery was significantly increased in patients presenting with bloody stool, in Arabs and those who were admitted to Hillel Yaffe Hospital.

**Conclusions:**

The incidence of intussusception prior to universal rotavirus immunization was documented in northern Israel. Despite the lower incidence, Arab patients underwent surgery more often, suggesting delayed hospital admission of Arab as opposed to Jewish patients.

## Background

Intussusception is among the most common abdominal emergencies among young children [1-4]. Symptoms include sudden onset of vomiting, abdominal pain, intermittent lethargy and irritability, and rectal bleeding that has been described as “currant jelly” [3-6]. Reduction is usually accomplished by air or barium enema, and in some cases by surgery, with or without bowel resection [3,4,6]. Intussusception primarily affects young children [3,5], with highest incidence in infants aged 4–10 months [3-5]. Reported yearly estimates of intussusception vary among populations and regions from 20 to 100 per 100,000 infants [3,7-9], but a higher incidence has also been reported [10].

The causes of intussusception are not fully understood, yet, there is evidence linking recent episodes of gastroenteritis and increased risk of intussusception [11,12]. Adenovirus was repeatedly recovered in higher proportions from fecal samples of patients with intussusception compared with control children [13-15], however no association has been found between natural rotavirus infection and intussusception [15-17].

In 1998 the reassortant rhesus human tetravalent oral rotavirus vaccine (RotaShield, Wyeth-Lederle, Pearl River, NY) was licensed in the United States. Shortly after its introduction into the routine childhood vaccination schedule, an excess risk for intussusception was found within 2 weeks after immunization with the first dose [1 intussusception case per 10,000 vaccinees [18-20]]. Consequently the vaccine was withdrawn from the market in 1999. Large clinical trials with two recent oral rotavirus vaccines (RotaTeq (Merck) [21] and Rotarix (GSK) [22]), and early post-marketing studies [23,24] showed no significant increase in post-immunization intussusception. However, later studies showed that in some settings e.g., Australia, and Mexico, there is an increased risk of intussusception during the first week post vaccination with the first dose of either rotavirus vaccine [25-27]. At present, this rare adverse event is estimated at about 1–2 intussusception cases per 100,000 vaccine recipients [28,29], nonetheless the vaccine’s benefits clearly exceed this small risk, thus rotavirus vaccines are recommended for use worldwide [29]. It is important to establish the baseline incidence of intussusception to assess the safety of rotavirus vaccines [3,29,30] in countries considering the introduction of rotavirus vaccination.

In Israel, rotavirus was found to be the most common pathogen causing acute gastroenteritis, and was detected in 39% of children less than 5 years of age hospitalized for diarrhea [31], leading to more than 4000 hospitalizations countrywide annually [31]. Both Rotarix and RotaTeq were licensed in Israel in mid-2007, but it was only in December 2010 that RotaTeq was included in the national immunization program. The aims of this study were to examine the incidence, clinical characteristics and potential correlates of intussusception among children less than five years of age from January 1^st^, 1992 to December 31^st^, 2009, before the introduction of rotavirus vaccine into the national immunization program.

## Methods

The study was conducted in two hospitals in northern Israel: Carmel in Haifa and Hillel Yaffe in Hadera. The population residing in the catchment area of the two hospitals includes representation of the two major ethnic groups of the Israeli population, Jews and Arabs. It is estimated that 20% and 90% of children aged 0–4 years in Haifa and Hadera sub-districts, respectively, receive inpatient services at these facilities. Based on this information and on publications of the Israel Central Bureau of Statistics the estimated number of children less than five years of age residing in the study area ranged from 29,000 in 1992 to 40,700 in 2009 (annual average 35,600).

We identified children less than five years of age who were hospitalized with intussusception (n = 190) at the study hospitals between January 1^st^, 1992 and December 31^st^, 2009 by searching for the ICD-9 diagnosis code for intussusception (560.0) in discharge records. All records with this code were retrieved regardless of its being a primary or secondary diagnosis. Also the word “intussusception” was searched in text regardless of diagnosis coding. In both hospitals, the diagnosis of intussusception was based on radiological findings, usually ultrasound. In order to examine the correlates of intussusception, we retrieved records of control children (N = 295) hospitalized for reasons other than intussusception. The primary diagnoses of the control children were trauma (50.3%), otitis media (23.8%), local infection (17%) (e.g., cellulitis, abscess, mastoiditis, urinary tract infection), fever (4.1%), and elective procedures/other (4.8%). From the archives of each hospital, we retrieved lists of potential consecutive control children with these diagnoses. Intussusception cases and control children were frequently matched by hospital, sex, season/date of admission (±2 months). We did not strictly match cases and controls by age; however knowing that the majority of cases were children under one year of age we restricted the age of controls to 24 months or less. Both case and control groups consisted of generally healthy children; 95% and 94% respectively had no underlying significant health problems. A control child with multiple hospitalizations was included only once.

Using a standardized form, demographic and clinical information was collected including age, sex, hospitalization date (month and year), maternal and paternal age, birth weight, birth week, history of gastroenteritis prior to hospitalization, breastfeeding, and significant medical problems. For cases, data were also obtained on clinical symptoms and treatment modality (e.g. air enema, barium enema or surgery). Socioeconomic rank of place of residence according to the Israel Bureau of Statistics [32] was used as a proxy measure of socioeconomic status; ranks 1–4, 5–6 and 7–10 were grouped as low, intermediate and high socioeconomic status, respectively.

The study protocol was approved by the institutional review board of Carmel medical center (Protocol number 0099-09-CMC) and Hillel Yaffe medical center (Protocol number HYMC-0051-09), which allowed access to medical records. Data abstraction was done by one person (S.E.), and the identity of the patients was kept confidential and was retained in the study hospitals.

### Sample size and power calculation

Assuming that the yearly incidence of intussusception in children less than five years of age is 35 per 100,000 children, with 95% confidence intervals (CIs) and maximum acceptable difference of 20 per 100,000 between the assumed and true incidence, then the required sample size was estimated at 33,602. The catchment area of the study hospitals had on average 35,600 children less than five years of age. Assuming a 30% prevalence of breastfeeding (i.e., 70% not breastfed), and an odds ratio (OR) for intussusception of 0.5 for children who are not breastfed, with the available 190 cases and 295 controls, we had 87% statistical power to detect a significant difference with two sided test.

### Statistical analysis

The average annual incidence (per 100,000) and 95% CIs of intussusception were calculated. Possible risk factors for intussusception and those for surgery were examined in intussusception patients, using Student t test for continuous variables and chi square test for categorical variables, and multivariable stepwise logistic regression models. The OR and 95% CI were obtained. The variables included in the analysis at step 1 were age, hospital, socioeconomic rank of place of residence, recent gastroenteritis episode, and breastfeeding. Two sided P < 0.05 was considered statistically significant. Data were analyzed with SPSS version 19.0. Imputation of missing values was not performed. Our research adhered to the STROBE guidelines (Additional file 1).

## Results

One hundred and ninety four hospitalizations for intussusception in 190 children between 01.01.1992 and 12.31.2009 were identified (4 children had recurrent episodes). Mean age of intussusception patients was 10 [Standard deviation (SD) 7] months; 65.3% were infants aged 3–11 months; 119 (62.6%) were boys. The most common symptom of intussusception was vomiting (77.3%), followed by irritability (69.1%), parental reporting of abdominal pain (47.8%), bloody stool (38.7%), lethargy (30.5%) and diarrhea (19.6%). Bloody stool was more common in infants 0–11 months of age than in children 12–59 months of age, 46.3% versus 19.6%, respectively, P < 0.001. Air enema, barium enema and surgery were performed in 62.4%, 33.5%, and 23.2% of the episodes, respectively. Median duration of hospital stay was 2 days (range 1 to 12).

**Figure 1:**
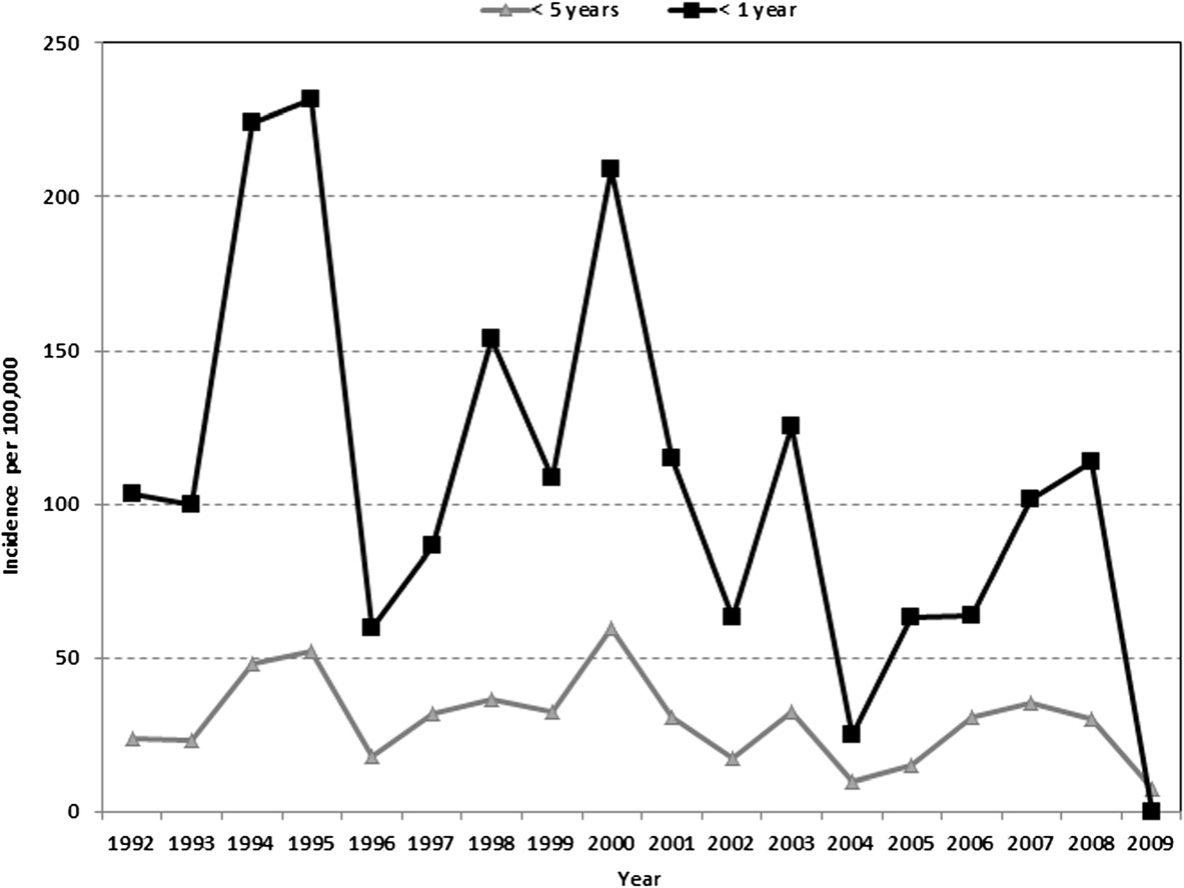
The incidence of intussusception (per 100,000) by age, in a northern region of Israel 1992–2009.

**Figure 2:**
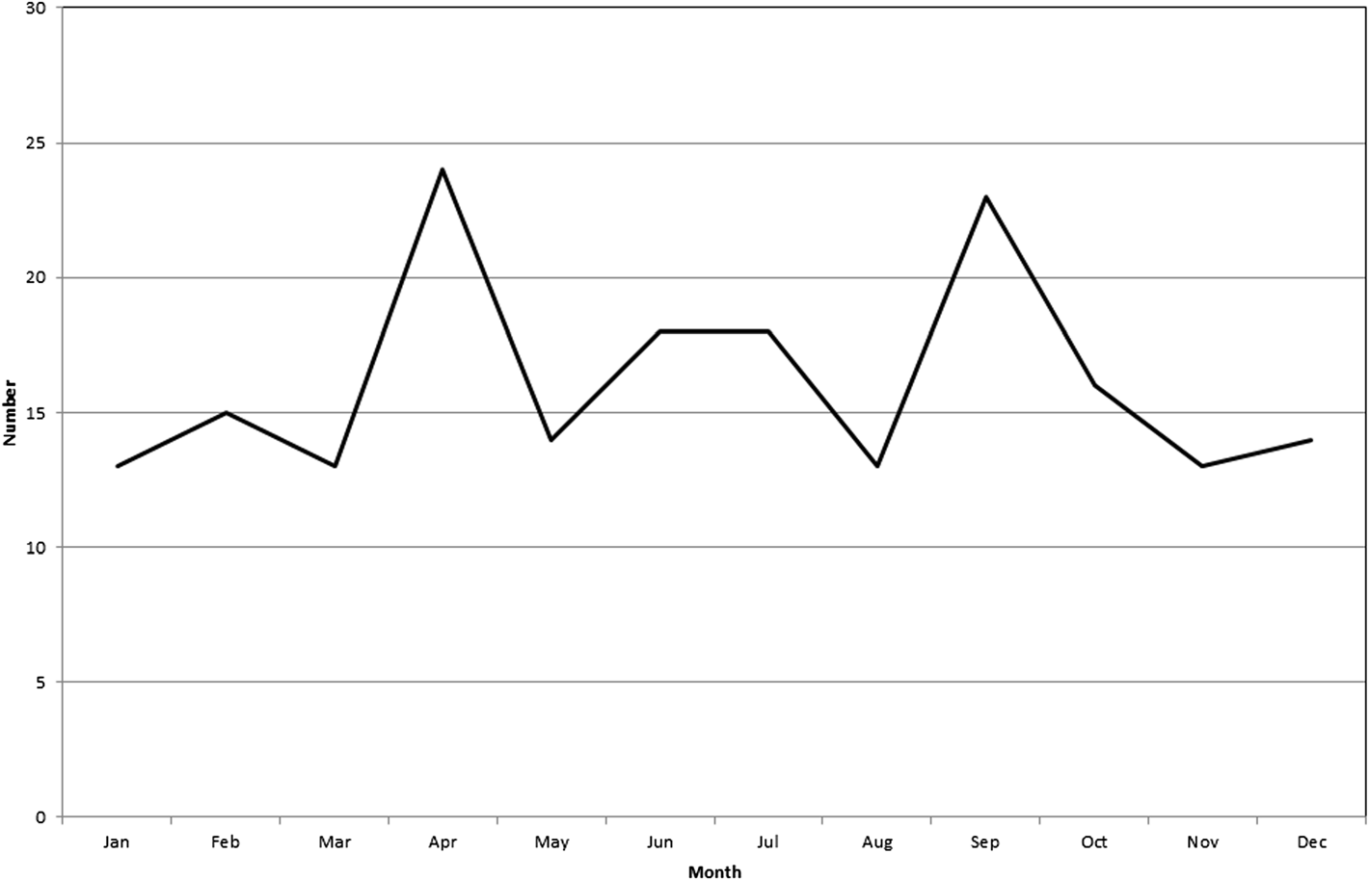
The number of intussusception episodes by month of admission in a northern region of Israel 1992–2009.

The overall annual incidence of intussusception was estimated at 29.5 (95% CI 16.4-53.1) per 100,000; 36.1 (95% CI 17.0-76.5) and 23.2 per 100,000 (95% CI 9.3-57.9) for Jewish and Arab children less than five years of age, respectively. The corresponding estimates for infants less than 12 months of age were 105.6 (95% CI 52.9-210.7) per 100,000; 128.1 (95% CI 53.0-309.6) and 80.1 (95% CI 29.1-242.6) per 100,000 in Jewish and Arab infants, respectively. There were year-to-year fluctuations in the incidence of intussusception, with a general trend of decline over the past few years (Figure 1). No clear evidence of seasonality was found (Figure 2). Children with intussusception and controls were similar in terms of sex, hospital, ethnic group and year and season of admission (Table 1). The percentages of children aged 3–5 and 6–11 months, those living in low socioeconomic settings, and children with history of gastroenteritis prior to hospitalization were significantly higher in cases than in controls (Table 2). Toddlers aged 1–4 years who were not breasted had a lesser likelihood of intussusception compared with breastfed children (Table 2). In multivariable analysis, associations with age, socioeconomic rank of town of residence, and recent gastroenteritis remained significant (Table 3). Since rotavirus vaccines were licensed and became available in Israel by mid-2007, an additional case–control analysis was performed including only children admitted prior to 2007. The results of both the bivariate and multivariable analyses were similar to the full dataset analysis presented.

**Table 1:** Distribution of cases and controls by year, season and sex

	**Cases, n (%)**	**Controls, n (%)**	**Pv**
	**(N = 190)**	**(N = 295)**	
**Year**			
1992	7 (3.7)	17 (5.8)	0.45
1993	7 (3.7)	9 (3.1)	
1994	15 (7.9)	24 (8.1)	
1995	17 (8.9)	17 (5.8)	
1996	6 (3.2)	15 (5.1)	
1997	11 (5.8)	15 (5.1)	
1998	12 (6.3)	15 (5.1)	
1999	12 (6.3)	25 (6.1)	
2000	22 (11.6)	36 (12.2)	
2001	12 (6.3)	20 (6.8)	
2002	7 (3.7)	7 (2.4)	
2003	11 (5.8)	24 (8.1)	
2004	4 (2.1)	11 (3.7)	
2005	6 (3.2)	14 (4.7)	
2006	12 (6.3)	16 (5.4)	
2007	14 (7.4)	19 (6.4)	
2008	12 (6.3)	4 (1.4)	
2009	3 (1.6)	4 (1.4)	
**Season**			
Winter	46 (24.2)	74 (25.1)	0.93
Spring	48 (25.3)	73 (24.7)	
Summer	48 (25.3)	68 (23.1)	
Fall	48 (25.3)	80 (27.1)	
**Sex**			
Males	119 (62.6)	176 (59.7)	0.54
Females	71 (37.4)	118 40.1)	
**Ethnic group by hospital**			
Hillel Yaffe Medical Center			
Arabs	49 (49.0)	56 (40.3)	
Jews	51 (51.0)	83 (59.7)	0.24
Carmel Medical Center			
Arabs	27 (30.0)	36 (23.2)	0.18
Jews	63 (70.0)	119 (76.8)	

**Table 2:** Bivariate analysis of the risk factors for intussusception

	**Cases**	**Controls**	**OR (95% CI)**	**Pv**
**Age (months), n (%)**				
< 3	10 (5.3)	33 (11.2)	Reference	<0.001
3-5	48 (25.3)	21 (7.1)	7.54 (3.14-18.07)	
6-11	76 (40.0)	88 (29.8)	2.85 (1.31-6.16)	0.008
12-23	44 (24.4)	136 (46.1)	1.06 (0.48-2.34)	0.87
24-59	12 (6.3)	17 (5.8)	2.32 (0.83-6.48)	0.10
Paternal age (yrs), mean (SD)^a^	34.7 (5.8)	33.3 (5.6)	1.04 (0.99-1.09)	0.078
Maternal age (yrs), mean (SD)^a^	31.0 (5.1)	29.6 (5.1)	1.05 (1.00-1.11)	0.045
SES of residence town, n (%)^a^				
Low	66 (42.9)	73 (29.4)	2.29 (1.32-3.97)	0.003
Intermediate	60 (39.0)	104 (41.9)	1.46 (0.85-2.51)	0.16
High	28 (18.2)	71 (28.6)	Reference	
Birth weight, n (%)				
Birth weight < 2500 gr^a^	6 (4.2)	14 (6.0)	Reference	0.44
Birth weight ≥ 2500 gr	138 (95.8)	221 (94.0)	1.45 (0.54-3.88)	
Birth week, n (%)				
Less than 37 weeks	6 (4.3)	10 (4.6)	Reference	0.86
≥ 37 weeks	135 (95.7)	206 (95.4)	1.09 (0.38-3.07)	
Gastroenteritis before admission, n (%)				
Yes	12 (6.3)	1 (0.3)	19.82 (2.55-153.72)	<0.001
No	178 (93.7)	294 (99.7)	Reference	
Breastfeeding < 1 yr, n (%)				
Breastfed aged < 1 yr	50 (37.3)	40 (28.2)	Reference	0.10
Not breasted children aged <1 yr	84 (62.7)	102 (71.8)	0.65 (0.39-1.09)	
Breastfeeding 1–4 yrs, n (%)				
Breastfed children age 1-4 yrs	9 (16.1)	8 (5.2)	Reference	
Not breasted children age 1-4 yrs	47 (83.9)	145 (94.8)	0.28 (0.10-0.78)	0.011

^a^Data on maternal and paternal age were available for 93 (47%) cases and 143 (48%) controls. Information on birth week was available for 141 (74%) cases and 216 (73%) controls, and on birth weight it was available for 144 (76%) cases and 235 (80%) controls. Information on SES of place of residence was available for 154 (81%) cases and 248 (84%) controls.

**Table 3:** Multivariable logistic regression model of the risk factors for intussusception in children less than 5 years of age

	Adjusted OR (95% CI)*	**Pv**
**Age (months)**		
<3	Reference	
3-5	5.30 (2.11-13.31)	<0.001
6-11	2.53 (1.13-5.62)	0.023
12-23	0.73 (0.32-1.65)	0.4
24-59	2.01 (0.73-5.99)	0.1
**Socioeconomic rank of place of residence**		
Low	2.81 (1.45-5.43)	0.002
Intermediate	1.66 (0.87-3.16)	0.1
High	Reference	0.12
**Gastroenteritis prior to hospitalization**		
No	Reference	
Yes	19.90 (2.35-168.32)	0.006

*Variables entered to the model at step 1: age, socioeconomic rank of place of residence, history of gastroenteritis, hospital, and breastfeeding. Data presented in the table are the final model which included the variables in the table and hospital.

Among patients with intussusception, the percentage of those who underwent surgery was significantly higher in infants, Arabs, residents of low socioeconomic status settings, in patients who had bloody stool and those admitted to Hillel Yaffe Medical Center (Table 4). In multivariable analysis the odds for surgery remained significantly 2-fold higher in patients who presented with bloody stool, in Arabs and in those who were admitted to Hillel Yaffe. Children who presented with diarrhea had lower odds for requiring surgery (Table 5).

**Table 4:** Correlates of surgery among cases with intussusception

	**Total**	**Surgery, n (%)**	**Pv**
Age			
< 1 year	134	36 (26.9)	0.11
1-4 years	56	9 (16.1)	
Sex			
Males	119	33 (27.7)	0.089
Females	71	12 (16.9)	
Ethnic group			
Arabs	76	25 (32.9)	0.015
Jews	114	20 (17.5)	
Socioeconomic rank of residence place			
Low	66	21 (31.8)	0.006
Intermediate	60	11 (18.3)	
High	28	2 (7.1)	
Hospital			
Hillel Yaffe Medical Center	100	33 (33.0)	0.001
Carmel Medical Center	90	12 (13.3)	
Breastfeeding			
Breastfed	59	17 (28.8)	0.26
Not breasted	131	28 (21.4)	
Blood in the stool			
No	117	22 (18.8)	0.045
Yes	73	23 (31.5)	
Vomiting			
No	44	6 (13.6)	0.074
Yes	146	39 (26.7)	
Diarrhea			
No	154	41 (26.6)	0.052
Yes	36	4 (11.1)	
Irritability			
No	59	17 (28.8)	0.26
Yes	131	28 (21.4)	

**Table 5:** Multivariable analysis of factors associated with surgery among patients with intussusception

	**Unadjusted OR (95% CI)**	Adjusted OR (95% CI)*	**Pv**
Hillel Yaffe Medical Center vs. Carmel Medical Center	3.20 (1.53-6.69)	4.42 (1.70-11.46)	0.002
Arabs vs. Jews	2.30 (1.16-4.54)	2.32 (0.99-5.45)	0.05
Blood in the stool (yes vs. no)	1.98 (1.00-3.91)	2.78 (1.17-6.60)	0.021
Diarrhea (yes vs. no)	0.34 (0.11-1.03)	0.22 (0.05-1.1)	0.06

*In addition to the variables in the table, the variables socioeconomic status of place of residence, age, sex, and interaction between ethnicity and hospital were added to the analysis. The adjusted ORs and Pv presented in the table were obtained from the final model.

## Discussion

We estimated the incidence and correlates of intussusception in northern Israel over 18 year period from 1992–2009, prior the introduction of universal rotavirus immunization in Israel.

The estimated mean annual incidence of intussusception among infants, 105.6 (95% CI 52.9-210.7) per 100,000 in this study, is slightly higher than the reported incidence in Europe [33-35], and about 2–3 fold higher than rates reported in the United States [7,36]. The incidence of intussusception in infants in this region of northern Israel decreased over time, in concert with previous studies [7,34,36,37].

The incidence of intussusception was higher among Jewish than Arab children, in agreement with an earlier study in southern Israel [10], where the incidence among Jewish and Bedouin children less than five year of age was estimated at 49.3 and 18.9 per 100,000, respectively, and for infants 199.6 and 66.8 per 100,000, respectively [10]. Ethnic differences in the incidence of intussusception have been described before [7,9,36-38]. Higher risk of intussusception has been noted among black and Hispanic children vs white, non-Hispanic children in the United States [7,36,38]. In Australia and New Zealand lower incidence of intussusception was observed in indigenous infants compared to non-indigenous infants [37], and specifically, among Maori compared with European infants, respectively [9].

About two thirds of intussusception cases occurred in infants 3–11 months of age; the risk of intussusception increased substantially by 5 fold in children aged 3–5 months compared to younger infants while children aged 6–11 months had about 2-fold increased risk of intussusception compared to the youngest age group (<3 months). Increased risk of intussusception was found more among children who lived in low socioeconomic communities than among those who lived high socioeconomic settings. A previous study from the United States showed that infants enrolled in Medicaid, used as a marker for low socioeconomic status, had 1.5 fold increased risk of intussusception [39]. It is not clear what underlying mechanisms might explain the association of intussusception and socioeconomic strata, but it is possible that genetic, environmental and cultural exposures including exposure to enteric pathogens and child nutritional practices [3] may play a role. In this study, recent history of gastroenteritis was associated with increased the risk of intussusception, and similar findings have been shown in other studies [11,12]. However, it is possible that our findings overestimate such an association, since physicians may have questioned parents of control children less intensively than intussusception patients’ parents on a recent history of gastroenteritis.

The common clinical symptoms of intussusception were similar to those reported previously; it is worth mentioning that visible (macroscopic) blood in stool was documented in only 39% of cases, and it appeared more than twofold in infants compared with toddlers. Irritability was also common, reported in 69%. This is probably due to the fact that infants and young children lack the ability to express pain verbally. These findings suggest that suspecting intussusception in children presenting with “atypical” symptoms is warranted.

The median hospital stay was 2 days, but reached 12 days in some cases. Usually, reduction with conservative treatment like air or barium enema was successful, but surgery was required in about 1/4 of intussusception patients. The percentage of intussusception patients undergoing surgery varied widely in previous studies - from 12% to 88% [3]. In our study, children who presented with bloody stool, Arabs, and those who were admitted to Hillel Yaffe Medical Center were more likely to undergo surgery. Despite the lower incidence of intussusception among Arab children, they underwent surgery about twice as often as did Jewish children. Interestingly a previous study from southern Israel also showed that Bedouin children with intussusception were more likely to undergo surgery than their Jewish peers [10]. However, in the southern Israel, about 50% of the Bedouin population lives in remote villages so that limited access to primary health care might explain why Bedouin children require surgery more often. This is not the case in northern Israel. In the study area, despite the fact that Arab residents live mostly in separate towns and villages, all have a basic infrastructure similar to that of Jewish communities, including on-site primary care clinics run by the main health maintenance organizations. Furthermore, in 1995 the National Health Insurance law was implemented in Israel, resulting in near uniform access to health care, preventive, ambulatory and inpatient services, thus minimizing disparities between Arab and Jewish populations. In the United States bowel resection was significantly increased in patients who had the symptoms for 2 days or more before admission compared to those who were admitted earlier [38]. Therefore, taking into account the characteristics of the study communities we postulate that admission may be delayed in some Arab patients with intussusception. This may be due to different referral behaviors of community physicians in Arab towns, parental perception of intussusception symptoms as non-serious (e.g., mistakenly confused with gastroenteritis), or both. If this hypothesis is proven true, the potential exists to reduce the need for intussusception surgery, especially in Arab children, by educating parents on when to seek medical care for young children with possible intussusception, and when pediatric caregivers in community practice should refer children to hospital for suspected intussusception. This finding may be relevant to countries with multiple ethnicities as well. Since mostly Arab physicians work in clinics in the Arab towns and villages, there is a possibility of a combined doctor-patient ethnic effect upon the decision whether or not to refer patients to hospital for further evaluation. Since the incidence of intussusception in Arab children is lower than that found among Jewish children, seeking medical attention might be delayed for intussusception with mild symptoms which might, in some cases, resolve spontaneously. This could lead to higher risk estimates for surgery among Arab vs. Jewish children. Therefore, the average incidence of intussusception associated with surgery was calculated, and was found to be higher among Arab vs. Jewish children (7.50 vs. 6.83 per 100,000 children less five years of age, but this difference was not significant).

The review of hospital records over an 18 year period from 2 hospitals in northern Israel yielded robust estimates of the incidence of intussusception, its clinical symptoms and treatment strategies prior the introduction of universal rotavirus immunization in Israel. The diagnosis of intussusception relied on radiological and/or sonographic findings throughout the study period in both medical centers. A case–control design was utilized to obtain insight in to the correlates of intussusception. These can be regarded as strengths of the study. Yet the study has some notable weaknesses: variability in obtaining clinical history probably occurred over time and among pediatricians. Hospital controls may not be the optimal control group, yet these groups were from the same source population and were comparable in terms of sex, study period, geographic region and ethnicity.

## Conclusions

We documented a relatively low incidence of pediatric intussusception prior the introduction of universal rotavirus immunization in Israel, but higher than that found in European and US children. Although incidence was lower among Arabs than Jews, the former group was more likely to undergo surgery, suggesting the possibility of delayed admission of Arab patients to hospital resulting from specific referral patterns of physicians and/or health care seeking behaviors of parents. These findings have public health and clinical implications.

## Abbreviations

CI: Confidence intervals; ICD-9: International classification of disease -9^th^ edition; SD: Standard deviation; OR: Odds ratio.

## Competing interests

The authors declare that they have no competing interests.

## Authors’ contributions

KM, ME, EK and DC contributed to the conception and design of the study. SE, SG, EK and ME made substantial contribution in data acquisition and analysis, and together with KM and DC they interpreted the study findings. KM and EK wrote the first draft of the manuscript, and DC and ME have been involved in significantly in critical revision of the article. All authors approved the final version of the manuscript.

## Pre-publication history

The pre-publication history for this paper can be accessed here:

http://www.biomedcentral.com/1471-2431/14/218/prepub

## Supplementary Material

Additional file 1 **STROBE Statement—Checklist of items that should be included in reports of *case-control studies.***
Click here for file
